# APIS—a novel approach for conditioning honey bees

**DOI:** 10.3389/fnbeh.2013.00029

**Published:** 2013-04-17

**Authors:** Nicholas H. Kirkerud, Henja-Niniane Wehmann, C. Giovanni Galizia, David Gustav

**Affiliations:** ^1^Department of Neurobiology, University of KonstanzKonstanz, Germany; ^2^International Max-Planck Research School for Organismal Biology, University of KonstanzKonstanz, Germany; ^3^Department of Electronic Engineering, University of RomeRome, Italy

**Keywords:** honey bee, behavior, automatic tracking, olfaction, automatic conditioning, aversive learning, short term memory, proboscis extension response

## Abstract

Honey bees perform robustly in different conditioning paradigms. This makes them excellent candidates for studying mechanisms of learning and memory at both an individual and a population level. Here we introduce a novel method of honey bee conditioning: APIS, the Automatic Performance Index System. In an enclosed walking arena where the interior is covered with an electric grid, presentation of odors from either end can be combined with weak electric shocks to form aversive associations. To quantify behavioral responses, we continuously monitor the movement of the bee by an automatic tracking system. We found that escapes from one side to the other, changes in velocity as well as distance and time spent away from the punished odor are suitable parameters to describe the bee's learning capabilities. Our data show that in a short-term memory test the response rate for the conditioned stimulus (CS) in APIS correlates well with response rate obtained from conventional Proboscis Extension Response (PER)-conditioning. Additionally, we discovered that bees modulate their behavior to aversively learned odors by reducing their rate, speed and magnitude of escapes and that both generalization and extinction seem to be different between appetitive and aversive stimuli. The advantages of this automatic system make it ideal for assessing learning rates in a standardized and convenient way, and its flexibility adds to the toolbox for studying honey bee behavior.

## Introduction

For almost 100 years, honey bees have been used as model organisms for the study of learning, memory, and the underlying neuronal substrates and mechanisms because they combine a rich behavioral repertoire with an easily accessible brain of ~1 mm^3^ in size (Menzel, 2001; Menzel and Giurfa, 2001; Chittka and Niven, 2009). The most frequently used method to date to investigate learning and memory in honey bees is the Proboscis Extension Response (PER)-paradigm: a conditioned stimulus (CS, which can be an odor, a tactile stimulus or light) is combined with an unconditioned stimulus (US, usually a sucrose reward), and the bee learns to associate the CS with the US, leading to an extension of the proboscis when the CS is given alone (Giurfa and Sandoz, 2012; Matsumoto et al., 2012). It has been shown in the fruit fly *Drosophila melanogaster* that there are major differences between appetitive and aversive conditioning, e.g., distinctive neuronal networks and biochemical pathways and different memory kinetics (Schwaerzel et al., 2003; Chabaud et al., 2006; Krashes and Waddell, 2008; Honjo and Furukubo-Tokunaga, 2009; Krashes et al., 2009; Cervantes-Sandoval and Davis, 2012). Therefore, it is important to establish also a potent method for aversive conditioning. To this end, several attempts have been made during the last 30 years, either in free-flying (or rarely free-running, Abramson et al., 1982; Abramson, 1986) or restrained honey bees (Vergoz et al., 2007; Carcaud et al., 2009; Giurfa et al., 2009; Mota et al., 2011). The concept of PER-conditioning was recently extended to aversive conditioning by introducing the sting extension response (SER)-paradigm: an olfactory or visual stimulus is paired with a mild electric shock (US), leading to an extension of the honey bee's sting (Vergoz et al., 2007; Mota et al., 2011). However, learning rates for honey bees undergoing the SER-conditioning remain low compared to the appetitive PER (see Vergoz et al., 2007 for SER, Carcaud et al., 2009 for comparison SER-PER). The comparably lower learning rates for aversive conditioning in honey bees and the simplicity of PER might explain why most studies focus on appetitive conditioning—despite the fact that aversive stimuli are as biologically relevant as appetitive ones: bees face different punishers in nature, such as beewolves (*Philanthus* spp.), social wasps and hornets, predacious bugs and spiders as well as conspecifics from other hives (Tinbergen, 1932; Butler and Free, 1951; Dukas, 2001; Dukas and Morse, 2003 and references therein; Ken et al., 2005; Abbott, 2006; Ings and Chittka, 2008; Abbott and Dukas, 2009; Nieh, 2010). From an ecological point of view, it is reasonable to assume that aversive memories are established more independently of the animal's internal state and—even more importantly—more readily consolidated and recalled as has been shown in *Drosophila*: a strong aversive memory is acquired after a single trial of electric shock (>100 V) reinforcement, whereas more appetitive training trials (at least two) with sugar reward are required to form an appetitive memory (Schwaerzel et al., 2003). Whether the same holds true for the honey bee as well remains elusive, because a systematic study comparing aversive and appetitive learning and memory in honey bees and possible differences in neuronal pathways and their molecular components has not been performed so far.

PER and SER have proven to be crucial in revealing basic concepts of learning and memory in the honey bee. Nevertheless, both methods have certain disadvantages: despite some efforts to automatize the conditioning (e.g., by Vareschi, 1971), both PER and SER involve considerable manual work because the bees need to be placed in special holders, anesthetized in order to allow their harnessing in these holders, and left in an undisturbed place for several hours before they can be used for conditioning. Additionally, during PER-conditioning the sucrose solution has to be administered to the bees by hand, and the bees' responses have to be recorded manually. Although harnessing of the bees allows stable physiological recordings combined with learning and retrieval tasks, it comes with the cost of considerable behavioral limitations. In contrast, most conditioning in *Drosophila* is done semi-automatically and with freely walking animals: the fruit flies are placed in a conditioning chamber, an electric shock is applied and the flies are conditioned and tested (Tully and Quinn, 1985; Schwaerzel et al., 2003; Claridge-Chang et al., 2009). This allows a high throughput of animals and comparison of different experiments in different laboratories becomes easier. Together with the molecular tools available, this has made *Drosophila* an important model species for studying mechanisms underlying learning and memory.

Because honey bees are the most prominent insect model system to investigate learning and memory due to their ability to solve cognitive-like tasks such as non-elemental forms of learning (for an overview, see Giurfa, 2003), we deem it necessary to develop a similarly standardized tool such as the one introduced by Claridge-Chang et al. (2009) for *Drosophila*.

In this paper, we present an automatic honey bee conditioning device (APIS—Automatic Performance Index System) together with a suitable protocol for aversive conditioning and an automatized analysis of honey bee learning and memory behavior. APIS uses freely moving animals instead of harnessed ones, allowing insight into natural decision making, reaction time, and response continuity as well as generalization and habituation. We show that the response rates to the CS achieved with this new device are comparable to the response rates of classical appetitive PER conditioning. Additionally, we are able to show that bees modulate their behavior with respect to the odors given and that both generalization and extinction seem to differ between PER and APIS. Our results suggest that APIS is a suitable tool to investigate learning and memory in honey bees, adding a new method to the toolbox and offering another opportunity to investigate possible differences between appetitive and aversive learning in insects.

## Materials and methods

### Honey bees

All experiments were conducted on *Apis mellifera* forager bees. The bees were caught either at feeders placed nearby the hives or when flying out at the entrance of the hives. With this approach bees of different age and foraging experience were randomly caught. This is likely to result in increased variations in individual learning capabilities compared to a more selective catching approach.

### PER—proboscis extension response

Once caught, the bees were immobilized using ice or CO_2_ and harnessed in small custom-built plastic holders similar to the ones described by Bitterman et al. (1983). After harnessing, the bees were kept in an undisturbed place for 2–3 h prior to the beginning of the experiment.

Ten minutes before the start of the experiment, each bee was checked for intact PER by slightly touching one antenna with 1.25 M sucrose solution without feeding the bee. Animals that did not show any response or could not move their mouthparts freely were excluded from the experiments.

An appetitive differential conditioning was used to analyse classical appetitive PER olfactory learning. One odor served as a reinforced CS (CS+), it was rewarded with 1.25 M sucrose solution (US); the other odor remained unrewarded (CS−, for a detailed description see Matsumoto et al., 2012). The odors were delivered to the honey bees using a custom-built, computer-controlled olfactometer. Air speed at the bees' head was about 0.9 m/s, generated by a carrier airstream and an odor stream. We avoided changes in air speed by compensating for the opening of the odor stream by closing a corresponding air stream. For all experiments, linalool (Merck Millipore, Darmstadt, Germany, 97% purity) and 1-nonanol (Sigma Aldrich, Taufkirchen, Germany, 98% purity) were used. 1-nonanol will henceforth be referred to as nonanol. Both odors were diluted 10^−3^ in mineral oil (Sigma-Aldrich). A total of 200 μl of the diluted odor was applied to rectangular Sugistrips (Kettenbach GmbH & Co. KG, Eschenburg, Germany) and placed at the distal end of 2 ml plastic syringes (Henke-Sass, Wolf GmbH, Tuttlingen, Germany) in the olfactometer. The accumulation of odors during the experiment was avoided by continuous air suction behind the conditioning apparatus. An inter-trial-interval (ITI) of 34 s was used throughout the experiments. Although it has been observed (in PER) that massed training with short ITI results in lower long-term memory learning rates (Menzel et al., 2001), we observed that freely walking bees in APIS tended to get exhausted when the protocol exceeded 20 minutes. Thus we shortened the ITI in both systems. The CS was presented to the bee for 4 s. Three seconds after onset of the CS+, the antennae were stimulated with the US, leading to a proboscis extension (thus, 1 s overlap of CS and US). The bee was allowed to feed for 3 s. In total, the bees were exposed to 8 odor stimuli during training, presented in a pseudorandomized order (ABBABAAB or BAABABBA) with either nonanol or linalool as odor A (CS+) in a balanced presentation. Five minutes after the last conditioning trial, bees were tested for their odor responses. The test procedure was similar to that for conditioning trials, except that no US was given after odor delivery. The test odors were applied in the order ABBA or BAAB in a balanced way.

During the experiment, the bee's response (PER or lack thereof) after the onset of the odor and before the presentation of the sugar water in the case of reinforced trials was recorded. Multiple responses during odor presentation were counted as a single PER. The response to the odor alone was noted as 1; no response or PER triggered by sugar water as well as the bee responding to the air stimulus prior to the odor stimulus was noted as 0.

### APIS—automatic performance index system

**Figure 1:**
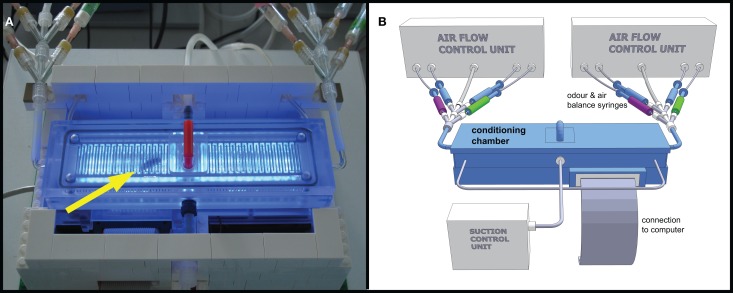
**Automatic Performance Index System APIS. (A)** Photograph of the conditioning chamber with honey bee inside (arrow). Blue LEDs activated to visualize bee and interior of chamber. Platform built in LEGO® containing cooling fans for controlling the temperature in the conditioning chamber and to embower the electronic circuit board beneath the chamber. Odor input tubes are visible in upper left and right corners. The bee was inserted into the chamber via the opening in the centre (lid with the red handle). The tubing on the backside shows parts of the suction, while the tube in the centre of the front shows a CO_2_ connection used for anesthesia during extraction of bee. **(B)** Schematic 3D model drawing of the APIS setup. The conditioning chamber consists of Makrolon® and is connected to a computer, controlling both stimulus administration via the odor syringes (green and magenta for the two odors, respectively, and blue for clean air) as well as recording the movements and actions of the bee inside the chamber. Influx and efflux of air into/out of the chamber were controlled throughout the experiment.

APIS consisted of a translucent acrylic glass (Makrolon®, Bayer MaterialScience, Leverkusen, Germany) conditioning chamber which was 148 mm long, 20 mm wide, and 6 mm deep (on the inside), enabling unhindered walking on either floor or ceiling for the honey bee. The interior surfaces of the chamber were covered with a metallic grid (1 mm wire width, 1 mm space between the wires, see Figure 1A) which could be electrified using a Grass SD 9 stimulator (Astro-Med GmbH, Rodgau, Germany). 26 infrared (IR) LEDs served as photo sensors, detecting and recording the position of the bee with a frequency of 5 Hz. Odors could be supplied at the narrow sides of the chamber via computer-controlled valves injecting odors into a constant airflow (Figure 1B). As in the PER conditioning, a change in air speed and amount as a result of the opening of the odor flow was compensated for by closing a corresponding air stream through otherwise open blank syringes (see Figure 1B). The air flow entering the chamber had a speed of 7.7 m/s, which decreased rapidly due to the construction of the chamber; at the center, the air speed was 0.8 m/s. To prevent odors from accumulating in the chamber and to facilitate odor distribution, the chamber was vented by suction for the entire duration of the experiment. The total airflow into the chamber was kept constant at ~1800 ml/min. The air inside was sucked out at three different points along the length of the chamber, with a total suction of ~1800 ml/min. Suction at the middle was 20% stronger than at each of the distal ends. The throughput of air per minute corresponds approximately to one hundred times the volume of the chamber. By that, we also tried to compensate for possible release of honey bees' alarm pheromone into the chamber. It is known that alarm pheromone decreases the learning ability of honey bees (Urlacher et al., 2010) and reduces the ability of bees to sense electric shocks (Núñez et al., 1997). The input and output air flow parameters were chosen as a consequence of the lowest obtainable flow rate that still gave clear electroantennogram (EAG) responses during a preliminary study (data not shown).

The odors were prepared in 2 ml plastic syringes as described for PER-conditioning above.

A customized device was built to catch and place the bee in the chamber without the use of anesthetics. After insertion bees almost immediately started to explore the arena by walking end to end.

Odor stimuli were set to 4 s and in case of the CS+ a mild electric shock (10 V) was administered to the bee 2 s after odor onset for the first trial (bee naïve to odor) and 1 s after odor onset for the next 3 trials (bee familiar with odor). The shock stimulus lasted for 3 s; the frequency was 1.2 pulses per second with a pulse duration of 200 ms. Thus, the bee received either one or two of the shock pulses overlapping with the odor stimulus (depending on whether it was the first CS+ or not). As in the PER conditioning, inter-trial-intervals (ITI) were set to 34 s and the bees were exposed to a total of 8 odor stimuli during conditioning, which were presented in a pseudorandomized order (e.g., ABBABAAB) starting with odor A or B in a balanced presentation and also balanced with respect to which side they were injected from. Both linalool and nonanol served as CS+ in a balanced way. During the training phase, the odors were introduced irrespective of the side at which the bee was located. Unfortunately, behavioral responses during the conditioning phase could not be quantified (see Results). During the testing phase, odors were delivered on the side the bee was located to give it the opportunity to withdraw from the odor. The bee's position was continuously sampled both during conditioning and test by the IR sensors and written to a log-file.

A customized script treated the acquired data in the following way: The movement trace of each stimulation period presented to the respective bee was extracted, and a bee crossing the middle without returning during the stimulation period was evaluated as “escape” (see Figure A2 for details). To compare the automatic tracking and quantification of responses with observable behavior during the recall phase, the bee's behavior was recorded by a human observer during the experiment. Avoidance of the odor was noted as 1, no response was noted down as a 0. After the experiment, the bee in the chamber was sedated by CO_2_ and sacrificed in 70% ethanol, and the interior of the chamber was cleaned with ethanol to remove possible pheromone marks and odor contamination.

### Statistical analysis

#### PER and escape responses

**Figure 2:**
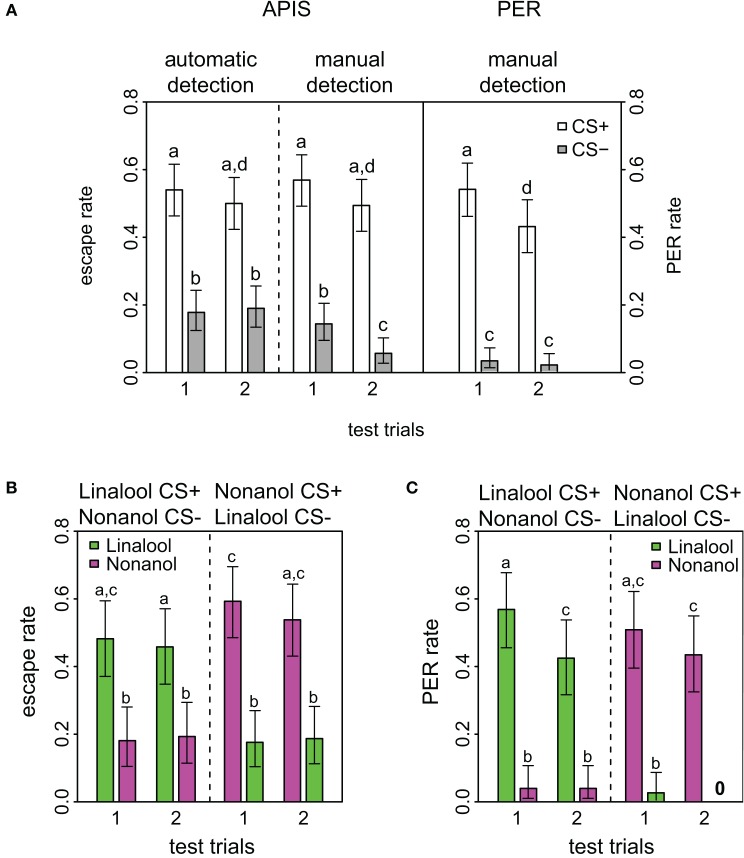
**Response rates in APIS and PER during short-term memory test. (A)** Observed response rates for APIS (left) and PER (right) during the four stimuli of the test trials. The left part of the figure depicts the high similarity between the automatically detected (far left) and the manually observed escapes (middle). Response rates to the CS+ are equal in both systems. Note as well the higher generalization levels for APIS, and the prominent extinction rate for PER. Error bars are 95% confidence interval. Groups that are significantly different (*p* < 0.05, by either proportion test or McNemar test) are indicated by different letters above the bars (*N* = 174 and 162 bees for APIS and PER, respectively). **(B)** Observed escape responses from APIS to the two odors linalool (green) and nonanol (magenta) during the four test trials of the recall phase. Left part of the plot contains the trials for the bees that experienced linalool as CS+ and nonanol as CS−, whereas the right side contains the trials for the bees that experienced nonanol as CS+ and linalool as CS− (*N* = 83 and *N* = 91, respectively). Response rates are higher for nonanol as CS+, while generalization is equal in both cases. **(C)** Directly observed responses from PER to the two odors during the four test trials of the recall phase (*N* = 82 in each group that received linalool or nonanol as CS+). Note the very low generalization for both groups, and the prominent extinction for linalool.

**Figure A1:**
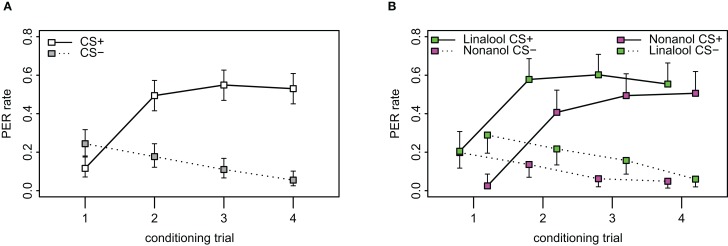
**Acquisition curves in PER. (A)** Observed PER rates during the 8 stimuli of the conditioning phase, 4 of which are responses to the CS+ (connected by solid line) and 4 to the CS− (dotted line) which were presented to the bees in pseudorandom order (ABBABAAB or BAABABBA). Difference between the responses to CS+ and CS− was significant from the second trial (*p* < 0.001, χ^2^ = 322.88, *df* = 8, repeated measurement logistic regression). *N* = 164. Error bars show 95% confidence intervals. **(B)** Bees were conditioned to linalool and nonanol, respectively. Left shifted values indicate the responses from the group of bees conditioned with linalool as CS+ and nonanol as CS−, while the right shifted values indicate the group that were conditioned with nonanol as CS+ and linalool as CS− (*N* = 82 for both groups). Linalool as CS+ was more easily learned than nonanol as CS+ (*p* < 0.001, χ^2^ = 39.8, *df* = 8, repeated measurement logistic regression with odor included as factor). Note also the higher spontaneous response for linalool as CS− for the first conditioning trial.

All analyses were performed using the open software R (R-Core-Team, 2012). The observed response rates in both conditioning and recall phase of PER, as well as manually and automatically obtained escape rates during recall phase of APIS were calculated together with the respective 95% Clopper-Pearson confidence intervals for the different stimuli groups (Figures 2, A1). PER responses during the conditioning phase were acquired for the period between odor onset and received sucrose reward (or a similar time period following CS− stimulation onset), whereas in the recall phase the presence of responses were evaluated within the entire odor stimulus periods (4 s) in both PER and APIS. Proportions tests were carried out to statistically compare rates across conditioning method (PER and APIS) and across odor stimulation protocol (linalool or nonanol as CS+), whereas McNemar Chi square tests were carried out for comparison between rates of different stimulus groups within each conditioning method and odor stimulation protocol. The response data acquired from PER during the training phase were fitted in a general linear mixed model by using the “glmer” function of the “lme4” package (Bates et al., 2011). The PER served as binary response variable, while trial, CS (CS+ or CS−) and odor (linalool or nonanol) with interactions were included as fixed effects. The bee identity served as random effect to account for the repeated measurements.

#### Velocity and attractance in APIS

For a more detailed analysis of the APIS data, we calculated velocity and an Attractance Index (AI) for each odor stimulus period in the recall phase.

**Figure A2:**
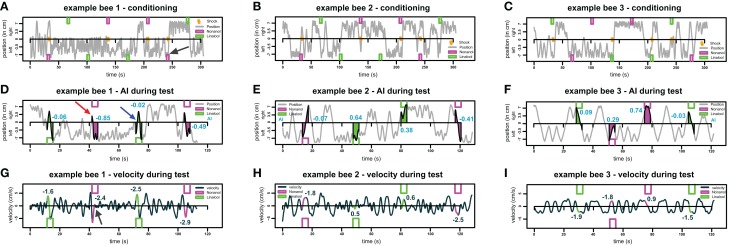
**Example traces for three different bees. (A–C)** Example movement traces for three different bees during the training phase. Bees moved in the conditioning chamber from left end (-7.4 cm) to right end (+7.4 cm). Green and magenta boxes indicate odor stimuli for linalool and nonanol, respectively. Orange zigzag lines indicate shock stimuli, the black arrow in **(A)** indicates an example response of a bee to shock: at the onset of the odor-shock-pairing, the bee oscillates on the left side and then escapes to the opposite side. **(D–F)** Movement of the same bee as in **(A–C)** during test phase. As in **(A–C)**, green and magenta boxes indicate odor stimuli for linalool and nonanol, respectively. AI is visualized by the colored polygons, and the blue numbers show the respective AI of each response. Crossing away from odor injection side without crossing back during stimulation period was counted as an escape. An example of an escape is indicated by the red arrow in **(D)**, the blue arrow indicates an example of a non-escape—the bee returns to the side it originated from. **(G–I)** Sea green line indicates velocity of same bees as in **A–F**, respectively, during the test. Colored parts of the line indicate the parts from which average velocity of each trial was calculated. The numbers indicate the respective velocity for each stimulus. Black arrow in **(G)** indicates an increase in velocity during odor presentation (escape), followed by local stasis.

**Figure A3:**
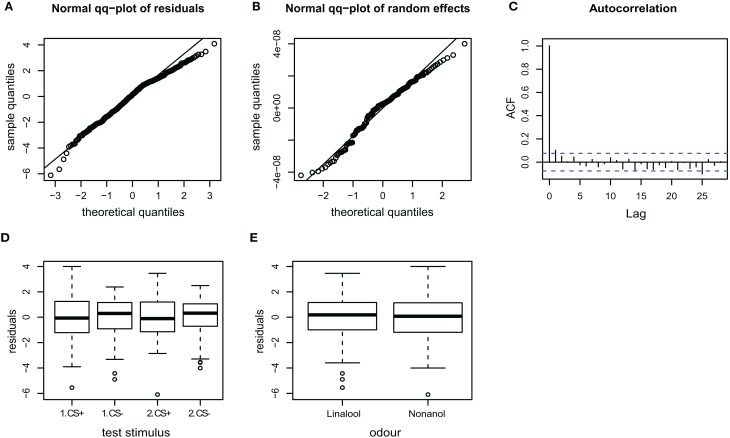
**Goodness of fit and diagnostic plots of residuals for linear mixed model used to predict velocity for APIS. (A)** Quantile probability plot showing the quantiles of the residuals of the observed velocity vs. the theoretical quantiles of a normal distribution. The velocity data mostly follows a normal distribution, but with a slightly heavy tail in the upper quantiles. This indicates that the upper values vary slightly less than when assuming an ideal normal distribution. **(B)** Quantile probability plot of the random effect (bee), indicating that the residuals of the velocity among the 174 tested bees follows a normal distribution. **(C)** Autocorrelation plot of the deviance residuals (first bar indicating the residual correlated with itself). The following bars each indicate correlation to the previous residual. Less than 5% of the correlation coefficients reach the significance levels (indicated by the broken blue lines), and no systematic pattern is evident. Thus no auto-correlation can be observed in the fitted data. **(D)** Boxplot of residuals of the velocity for the test trials indicates that the residuals within each testing group are symmetrically distributed around the predicted means (which are aligned to *y* = 0). The residual variation seems to be slightly smaller for the CS− than the CS+ test trials, indicating that velocities following the odor that was paired with shock during the training phase vary to a greater extent than to the velocities following non-shock-paired odor. **(E)** Residuals plotted against the odors used for stimulation. The residuals of the velocity vary to similar degrees for both linalool and nonanol.

The velocity (in cm/s) for each stimulus was calculated during the first 2 s following odor onset by assessing the time spent at each position of the movement trace (Δt) and fitting a cubic spline function. Movement away from the odor injection was assessed as negative velocity and movement toward it as positive velocity (see Figures A2G–I in Appendix). Velocity data were fitted by a linear mixed model with stimulus (1.CS+, 2.CS+, 1.CS−, 2.CS−) and odor (linalool, nonanol) as predictor variables and bee as random effect to account for the repeated measurements. The “lmer” function in the “lme4” package was used for fitting the model. With the “sim” function from the “arm” package (Gelman et al., 2012) one thousand different outcomes of the model parameters were simulated, thus creating the predictive posterior distribution. Fitted mean velocity for each stimulus type was calculated with the “fixef” function and 95% credible intervals were calculated from the simulated parameters of the predictive posterior distribution (see Figure A3 in Appendix for goodness of fit and residual analysis).

To quantify the bee's movement with respect to the odor injection, the integral of the movement trace was calculated following each odor stimulus. Since the integral comprises both distance and time spent away from the injected odor, it can be viewed as the magnitude of the odor response.

**Figure A4:**
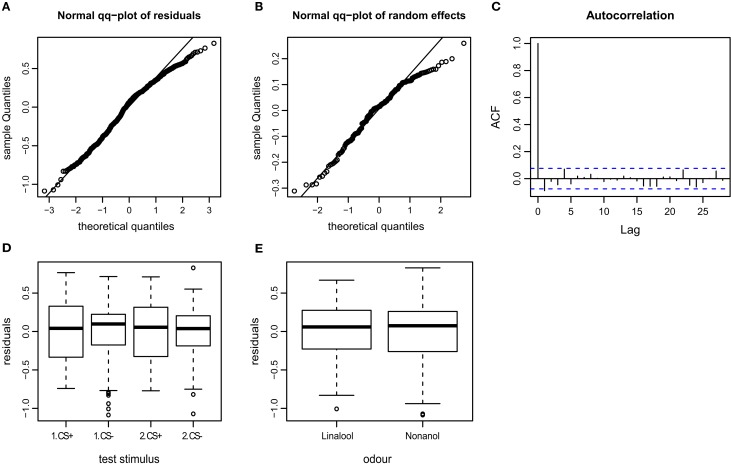
**Goodness of fit and diagnostic plots of residuals for linear mixed model used to predict AI (Attraction Index) for APIS. (A)** Quantile probability plot showing the quantiles of the residuals of the observed AI vs. the theoretical quantiles of a normal distribution. The AI data follows a normal distribution, except for a slightly heavy tail in the upper quantiles (as can be observed for the velocity). This indicates that the upper values vary less than when assuming an ideal normal distribution. **(B)** Quantile probability plot of the random effect (bee), indicating that the residuals of the AI among the 174 tested bees mostly follows a normal distribution, but with a distinct heavy upper tail. **(C)** Autocorrelation plot of the deviance residuals (first bar indicating the residual correlated with itself). The following bars each indicate correlation to the previous residual. Less than 5% of the correlation coefficients reach the significance levels (indicated by the broken blue lines), and no systematic pattern is evident. Thus no auto-correlation can be observed in the fitted data. **(D)** Boxplot of residuals of the AI for the test trials indicates that the residuals within each testing group are symmetrically distributed around the predicted means (which are aligned to y = 0). The residual variation seems to be smaller for the CS− than the CS+ test trials, indicating that AI following the odor that was paired with shock during the training phase vary to a greater extent than the AI following non-shock-paired odor. **(E)** Residuals plotted against the odors used for stimulation. The residuals of the AI vary to similar degrees for both linalool and nonanol.

Attractance Index for each stimulus was calculated as:

$$AI_{\text{stim}}=SI\int_{t_{\text{stim}}}^{t_{\text{stim}}\text{+4s}}P(t)dt$$

where *t* (time) is given in seconds, and *t*_stim_ is the time of onset for the respective odor stimulus. *P*(*t*) is the position at time *t* ranging from −7.4 cm (left end) to +7.4 cm (right end of the chamber). The integral was approximated with the trapezoid rule (see Figures A2 D–F in Appendix for examples) and multiplied by the side index *SI* which indicates the side of odor injection for the respective stimulus. The AI values of each stimulus were then normalized with respect to the minimum and maximum observed integral of the whole population of the tested bees so that the final AI values were dimensionless (without units) and ranged from −1 to 1. Since the bees were located on the side where the odor was injected at the time of stimulus onset, the AI had a positive bias. Therefore avoidance did not necessarily result in a negative AI. The AI data were fitted by a linear mixed model similarly to the velocity data as mentioned above (see Figure A4 in Appendix for goodness of fit and residual analysis). A reduced variance for the higher AI values (as seen in Figures A4A,B, where the higher ends of the residuals are slightly lower than when considering an ideal normal distribution) can be explained by the observation that the attraction response among the bees to an odor in the conditioning chamber is more homogenous than the avoidance response. This is also the case for velocity (see Figures A3A,B).

All *p*-values mentioned for the velocity and AI data were obtained by calculating the probability of a simulated parameter from one group being within the range of the 95% credible interval of the compared group. Since no informative priors have been implemented in the respective models, the fitted means resemble the arithmetic means, and the credible intervals resemble the confidence intervals of the observed data.

## Results

### PER-conditioning

To assess the quality of our new learning paradigm, we conditioned honey bees with the standard PER-paradigm for comparison. A total of 209 bees were caught in order to condition them, of which 164 bees (78.5%) were used for conditioning while 45 died before or during the experiment or were unable to extend the proboscis upon the presentation of sugar water prior to conditioning. Bees were conditioned with an eight-trial differential conditioning paradigm. Acquisition during conditioning followed the typical curve for differential conditioning (Figure A1A in Appendix) as has been observed for differential classical conditioning across animal species, experimental paradigms and research labs. Bees' responses to the CS+ increased significantly during trials (*p* < 0.001, χ^2^ = 179.3, *df* = 6, repeated measurement logistic regression), whereas it decreased over trials for the CS− (*p* < 0.001, χ^2^ = 38.8, *df* = 6). We observed a spontaneous response to the first odor stimulation (12.3%) which is common for PER-conditioning (Menzel, 1990). We also observed a generalization effect, where bees were more likely to respond to the first CS− (32.9%) when it followed a CS+, than to the first CS+ (14.6%) when it followed a CS− (*p* = 0.006, χ^2^ = 7.6, *df* = 1, data not shown). From the second trial on, the bees successfully distinguished between the CS+ and the CS− (*p* < 0.001, χ^2^ = 322.9, *df* = 8), and the response rate stabilized after three trials of the CS+. For the last training trial, 53.0% of bees responded to the CS+, whereas only 6.5% of the animals responded to the CS−.

#### Effect of odor on learning performance in PER

Of the 164 bees that underwent the appetitive PER conditioning, 82 were conditioned with nonanol as CS+ (linalool as CS−), and 82 with linalool as the CS+ (nonanol as CS−). We found that the odor choice matters: linalool as CS+ (left-shifted series in Figure A1B) was more likely to elicit a response than nonanol as CS+ (right-shifted series in Figure A1B; *p* = 0.001, χ^2^ = 18.3, *df* = 4). The same was observed for the CS− (dotted lines) (*p* = 0.048, χ^2^ = 9.6, *df* = 4, repeated measurement logistic regression). Inspected in more detail, the difference between the CS+ responses to the respective odors was significant for the first (*p* < 0.001, χ^2^ = 12.9, *df* = 1, proportions test) and marginally for the second trial (*p* = 0.028, χ^2^ = 4.8, *df* = 1), but not for the third (*p* = 0.162, χ^2^ = 1.9, *df* = 1) and fourth (*p* = 0.54, χ^2^ = 0.4, *df* = 1). Thus, nonanol needed one more trial to reach the same acquisition level as linalool. However, there were no significant differences between responses to the CS− for the individual trials.

We observed both asymmetric generalization and differences in the spontaneous responses to the odors. When linalool served as CS− and followed nonanol as CS+ during the first training trial, 38.1% of the bees responded to linalool. In contrast, when nonanol served as CS− and followed linalool, only 29.3% of the bees responded (data not shown). Regarding the spontaneous response to one of the odors, nonanol was much less likely to elicit a PER during the very first presentation of the odor: only 6.0% of the bees responded to nonanol, whereas 18.3% of the bees responded with a PER to the very first presentation of linalool (data not shown). Taken together with the slower learning of nonanol this indicates that linalool has a higher hedonic value than nonanol, leading to drastically increased spontaneous responses and marginally elevated generalization in cases where nonanol was CS+ and followed by linalool as CS−.

#### Effect of anesthesia on learning performance in PER

For PER-conditioning, different anesthetizing methods were used: 82 of the bees were anesthetized with ice and 82 with CO_2_. For the fourth trial, there was a marginal significant difference in response between the two groups: bees anesthetized with ice responded with 59.5%, whereas bees anesthetized with CO_2_ responded only with 46.2% (*p* = 0.045, χ^2^ = 4.0, *df* = 1 Figure A9A). For the first trial, the response rate to the CS− was significantly higher than to the CS+ in CO_2_-anesthetized bees only (*p* = 0.009, χ^2^ = 6.72, *df* = 1). The difference detected in the fourth trial indicates that CO_2_ has a slight negative influence on the acquisition of the bees compared to ice, which could be a result of a negative side-effect of this type of anesthesia on the learning process at a molecular level. Indeed it has been previously shown that both ice and CO_2_ affect the bees' behavior (Pankiw and Page, 2003; Frost et al., 2011). For the recall phase, the reduction in response rate from the first to the second CS+ is consistent with the observations made when not taking anesthesia into consideration (compare Figure A9B with PER rates in Figure 2A). Taken together with the observation that no significant differences across stimulus groups of the two anesthesia application methods were detected this implies that the method of anesthesia has no effect on the short-term memory of the honey bees.

### Conditioning in APIS

With the APIS, 192 bees were conditioned in the course of this experiment. In total 174 (90.6%) of these were analysed, the remaining 19 bees were discarded either due to exhaustion (bees stopped moving during the experiment) or due to technical difficulties during conditioning and/or during testing. Of the 174 bees tested, 91 were conditioned using nonanol as CS+, and 83 were conditioned with linalool as CS+.

The bees introduced to the chamber almost immediately started exploring it (see Figure 1). The bees usually explored both sides of the chamber prior to the onset of odors, without any noticeable impairment of their walking behavior.

Bees usually responded with a jump when receiving an electric shock. Following this, most of the bees increased walking speed (see below) and sometimes started intense buzzing, a behavior not displayed in the absence of electric shock. After some training trials, buzzing and quick turning as well as running away from the injected odor could be observed at the onset of the CS+, showing that the bees detected the odor and associated it with the punishment. The mild electric shock of 10 V seemed not to harm the bees, since none of the animals showed signs of impairment through the shock. These results are in line with the observations reported by other groups that have conditioned bees with electric shocks (Vergoz et al., 2007; Agarwal et al., 2011).

We decided to give the shocks in short pulses instead of one continuous pulse because we observed that this is less intense for the bees, but still gives a high rate of responses (>95% of the tested bees responded visibly to two or more of the 10 V shock pulses of 200 ms). Interestingly, we also observed that some of the bees did not react to the first one or two of the 200 ms shock pulses although they were in contact with the grid, suggesting that there are individual differences in shock susceptibility. Nevertheless, we observed that electric shocks had a cumulative effect, where the last pulse generally resulted in a stronger response than the first one.

In the version of APIS used for this study, we could not obtain acquisition rates, because of the low sampling frequency of 5 Hz and because the onset of the odor could not be exactly determined with respect to the honey bees' position in the apparatus. This also prevented us from using the longer ISI of the first trial (2 s) to analyze the innate reaction of the bees to the odors, although we observed a general tendency of naïve bees approaching the odor. In future versions of APIS we will increase the sampling frequency, hopefully allowing a more detailed analysis of the bees' behavior during the acquisition period.

### Short-term memory performances in APIS and PER

#### Assessing the data quality in APIS

Short-term memory was tested 5 min after conditioning in both systems. For this testing phase all APIS data were automatically acquired as described in the methods. Manual observation and evaluation of escapes were also performed, and the comparison between the automatically and manually acquired escapes had an overall concordance of 86.1%. This describes the percentage of odor response ratings that were the same for both methods. Such a low discrepancy substantiates both that the experimenters were largely accurate in their evaluations of escapes and that the automatic quantification script was suitable. However, there was one significant and striking difference between automatic and manual observation: for the last CS−, the manual detection reported only half of the escapes that were determined by the automatic tracking system (Figure 2A, fourth gray bar). We assume that this difference reflected the expectancy of the human observers that bees will not escape on the second CS−, because less bees escaped from the CS− than the CS+ during the first part of the test and observers might have anticipated that bees in the absence of shock show signs of extinction (see below).

#### Comparison of response rates APIS-PER

Response to the CS+ was significantly higher than to the CS− for both conditioning methods (APIS: *p* < 0.001, χ^2^ = 49.6, *df* = 1; PER: *p* < 0.001, χ^2^ = 103.4, *df* = 1) and over both trials (APIS: *p* < 0.001, χ^2^ = 37.1, *df* = 1; PER: *p* < 0.001, χ^2^= 79.1, *df* = 1), indicating that honey bees learned the correct associations in both the appetitive setting of the PER and the aversive setting of the APIS (Figure 2). In APIS, the escape rate for the first CS+ was 54.0% (first white bar in Figure 2) and for the first CS− it was 17.8% (first gray bar). PER rate for the first CS+ was 53.7% (fifth white bar), whereas to the first CS− it was 3.0% (fifth gray bar). This approximate 6-fold difference between the CS− responses (17.8 vs. 3.0%) of the two systems suggests that the extent of generalization is higher for the aversively trained bees in APIS than for the bees appetitively conditioned with PER. Reduction in response rate from the first to the second trial is relevant for quantifying extinction by odor exposure only: reduction in response to the CS+ was significant in PER (*p* < 0.001, χ^2^ = 12.0, *df* = 1), but not in APIS (*p* = 0.488, χ^2^ = 0.48, *df* = 1).

#### Velocity and magnitude of odor responses in APIS

**Figure 3:**
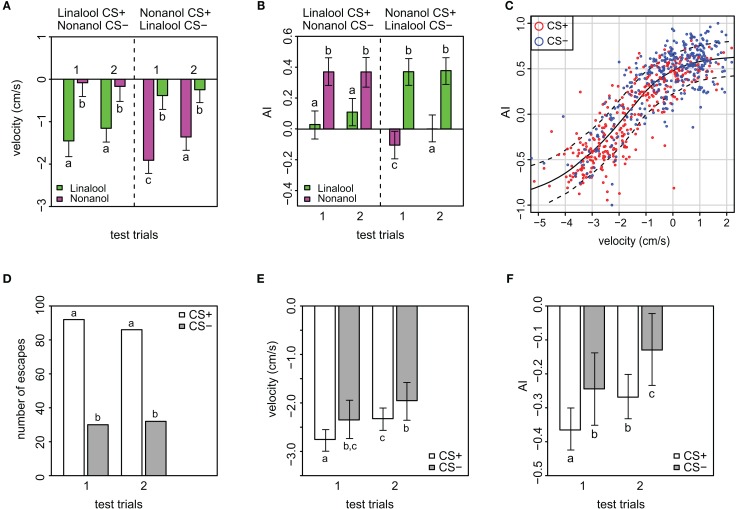
**Velocity and magnitude of odor response (AI) in APIS. (A)** Velocity toward (positive) or away from (negative) the introduced odor in the conditioning chamber during the two first seconds of the stimuli in the test phase. Note the low generalization for this parameter, and that extinction is only evident in nonanol conditioned bees. Additionally, velocity away from the odor is higher for nonanol than for linalool. Error bars indicate 95% credible intervals. Groups that are significantly different (*p* < 0.05, by proportion of simulated parameters from one group occurring within the credible interval of the compared group) are indicated by different letters above the bars (*N* = 83 and 91 bees that received either linalool or nonanol as CS+, respectively). See Figure A3 for goodness of fit. **(B)** Magnitude of odor responses represented by the mean Attractance Index (AI) for the different stimuli of the test phase in APIS. Note the low generalization, and that AI reveals extinction only in the nonanol conditioned bees. Error bars indicate 95% credible intervals. Groups that are significantly different (*p* < 0.05) are indicated by different letters above the bars (*N* = 83 and *N* = 91 bees that received either linalool or nonanol as CS+, respectively). See Figure A4 for goodness of fit. **(C)** AI plotted against velocity, red: CS+, blue: CS−. Loess smoothing (solid line) and residual spread (broken lines). The analysis shows a strong relationship between the two parameters. **(D)** Numbers of escapes for the different stimuli indicating the subset used for the analyses showed in the following panels **(E,F)**. **(E)** Analysis of all escape responses with respect to the bees' velocity, stratified by CS+ and CS−. Note that bees escaped with a higher speed from the CS+ than from the CS−, indicating that even when escaping, some information about the associative significance of the odor was present. Additionally, the velocity is less negative for the second than the first CS+, indicating extinction. Error bars are 95% credible intervals, while different letters indicate statistically significant different groups. See Figure A5 for goodness of fit. **(F)** Analysis of AI responses, stratified by CS+ and CS− for escapes only. Note that AI score was higher for the CS+ than from the CS−, confirming the data on velocity (Figure 3E): even though the bees escape wrongly to the CS−, there is some information about the associative significance of the odor present, modulating the bees' behavior. As for velocity, the AI is less negative for the second CS+. Error bars are 95% credible intervals, and different letters indicate statistically significantly different groups. See Figure A6 for goodness of fit and residual analysis.

When looking at the bees' movement responses during recall in more detail, i.e., velocity and AI, clear differences between CS+ and CS− were apparent. Velocity changed drastically during an escape: for the CS−, the average velocity over the two trials was −0.2 cm/s while for the CS+ it was −1.5 cm/s. This significant change in velocity (*p* < 0.001, see Materials and Methods for statistics) indicates that bees escaped with higher speed away from the odor that was paired with shock in the previous conditioning phase (Figure 3A). When taking into account the different effects of the odor identity, the velocity was more negative for nonanol than for linalool as CS+ (*p* = 0.031). Additionally, when comparing the first and the second CS+ stimulus, the velocity was more negative for the first (*p* = 0.008), but only in the case where nonanol was acting as the CS+. The Spearman's rank correlation between velocity and escape response is −0.73 (±0.03), thus a successful escape tends to result in a more negative velocity.

The AI reflects the magnitude of the avoidance or attraction by taking the approximate integral of the movement trace for each stimulation period (see Figures A2D–F for examples). For both test trials, the AI of the CS+ was significantly lower than for the CS− (*p* < 0.001), indicating a clear avoidance from the shock-paired odor (Figure 3B). Generally, an AI of 0.3 or higher could be regarded as attraction, whereas an AI of 0.1 or lower was typical for avoidance. This is reflected when looking at the AI-distribution with respect to the CS+ and CS− (Figure A8). In accordance with the escape rate results and the results for velocity, the AI of the first CS+ was lower than that of the second CS+ stimulus for nonanol (*p* = 0.023), but not for linalool (*p* = 0.097). For the responses to the CS−, no such differences were apparent between the two test trials. The correlation between AI and escape response is −0.76 (±0.03), thus a successfully escaping bee is more likely to incur a lower AI. Interestingly, there was a difference between nonanol and linalool detectable in AI and velocity which was not apparent when looking at the escapes: there is an elevated (meaning: more negative) escape velocity for nonanol as CS+ than for linalool (Figure 3A, *p* = 0.031). Similarly for the AI, nonanol as CS+ elicits a significantly more negative AI compared to linalool as CS+ for the first test trial (Figure 3B, *p* = 0.022), and marginally non-significant for the second trial (*p* = 0.054). These results might be explained by the observation made for the PER conditioning: bees seem to have an innate appetitive preference to linalool (Figure A1B), leading to an increased chance of response. Similarly, linalool might act as an attractant (resulting in decreased velocity away from the odor, Figure 3A) in APIS during the test.

The strong correlation between velocity and AI (0.84 ± 0.02) is expected since the higher the speed with which the bees escape, the greater the magnitude of their escapes is likely to be. Plotting velocity against AI reveals an increase in positive velocity (toward odor), reflected by an asymptotic increase in AI toward a maximum of 1. Additionally, an increase in negative velocity (away from odor) results in a decrease in the AI asymptotically toward a minimum of −1 (Figure 3C). The responses to the CS+ generally make up the lower end of the sigmoidal curve, while the responses to CS− make up the higher end (red and blue circles in Figure 3C, respectively). This sigmoidal relation between AI and velocity is left-shifted with respect to 0 on the velocity axis (inflection point at −2), which implicates that higher negative speed is more prominent than high positive speed during the 2 s following odor onset.

**Figure A5:**
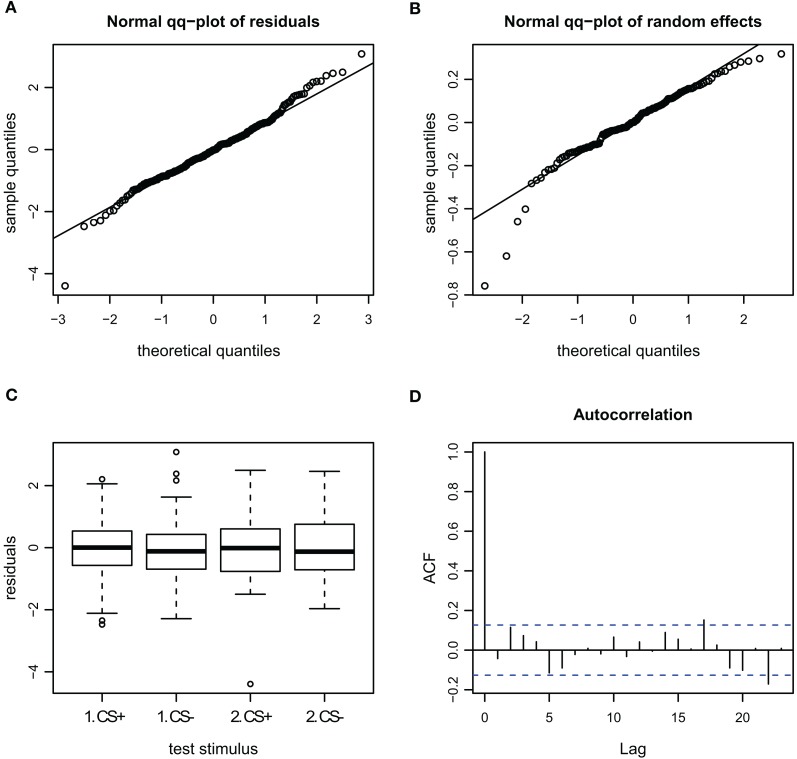
**Goodness of fit and diagnostic plots of residuals for linear mixed model for the velocity during escape in APIS. (A)** Quantile probability plot showing the quantiles of the residuals of observed velocity vs. the theoretical quantiles of a normal distribution. The observed velocity of the escape subset follows a normal distribution, and the heavy tail for the upper quantiles that was apparent in the full dataset (Figure A3) is gone. This indicates that none-escape responses were responsible for the low variation. **(B)** Quantile probability plot of the random effect (bee), indicating that the residuals of the velocity among the tested bees in the escape subset follows a normal distribution. **(C)** Boxplot of residuals of the velocity for the test trials from the escape subset indicates that the residuals within each testing group are symmetrically distributed around the predicted means (which are aligned to y = 0). **(D)** Autocorrelation plot of the deviance residuals (first bar indicating the residual correlated with itself). The following bars each indicate correlation to the previous residual. No auto-correlation can be observed in the fitted data.

**Figure A6:**
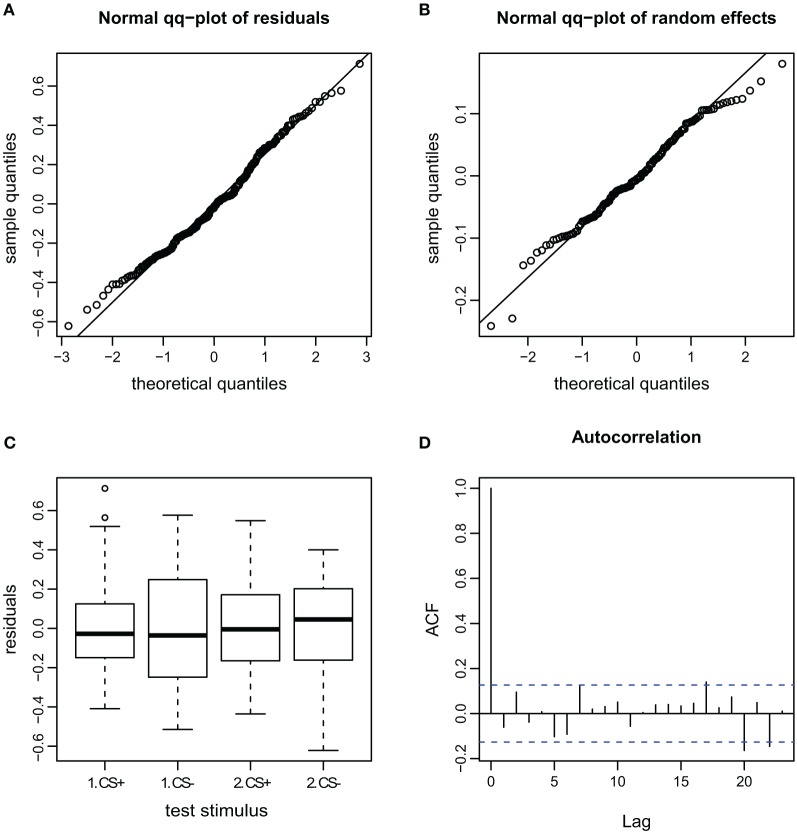
**Goodness of fit and diagnostic plots of residuals for linear mixed model for the AI during escape in APIS. (A)** Quantile probability plot showing the quantiles of the residuals of observed AI vs. the theoretical quantiles of a normal distribution. The observed AI of the escape subset follows a normal distribution, and the heavy tail for the upper quantiles that was apparent in the full dataset (Figure A4) is gone. This indicates that none-escape responses were responsible for the low variation (as in the velocity observations seen in Figure A5). **(B)** Quantile probability plot of the random effect (bee), indicating that the residuals of the AI among the tested bees in the escape subset follows a normal distribution. **(C)** Boxplot of residuals of the AI for the test trials from the escape subset indicates that the residuals within each testing group are symmetrically distributed around the predicted means (which are aligned to y = 0). Variance of the residuals seems to be lower for the CS+ than the CS− stimuli (especially for the first trial), indicating that AI in escaping bees varied less for the first CS+ stimulus than for the remaining stimuli. **(D)** Autocorrelation plot of the deviance residuals (first bar indicating the residual correlated with itself). The following bars each indicate correlation to the previous residual.

We were curious whether bees that escaped from the odors showed a rather stereotypic escape response or whether they somehow modulate their behavior according to the stimulus presented during the test. Thus we analysed a subset of the data consisting of only escape responses for possible differences in velocity and AI (Figures 3D–F and Figures A5, A6 for model fit and residual analysis). The number of escapes from the CS+ was about three times higher than escapes from the CS−, reflecting the differences in response rates as previously mentioned (Figure 3D). As expected, both the velocity and AI average values are generally more negative when excluding the non-escapes (Figures 3E,F). More interesting however is that both the velocity and the AI of the bees that “correctly” escaped to the CS+ are more negative than for the bees that “wrongly” escaped to the CS− (velocity: *p* = 0.038, AI: *p* = 0.027), indicating that the bees modified their behavior to the respective stimuli despite escaping. In addition, the bees reduce the velocity and magnitude of escape from the first to the second CS+ (Figures 3E,F), which indicates a subtle extinction not detectable when considering the presence of escape alone (Figures 2A,B).

Whereas in the dataset with all responses included the average velocity for CS+ was −1.5 cm/s, the same average in the escape subset was almost twice as negative (−2.5 cm/s). Similarly, the AI for the CS+ responses was reduced from an average of 0 to an average of −0.32 and from 0.37 to −0.187 for the CS− when considering escapes alone. The differences found within the escape subset argue for the use of the continuous variables (velocity and AI) as being more suitable for quantification of distinct behavioral changes.

**Figure A7:**
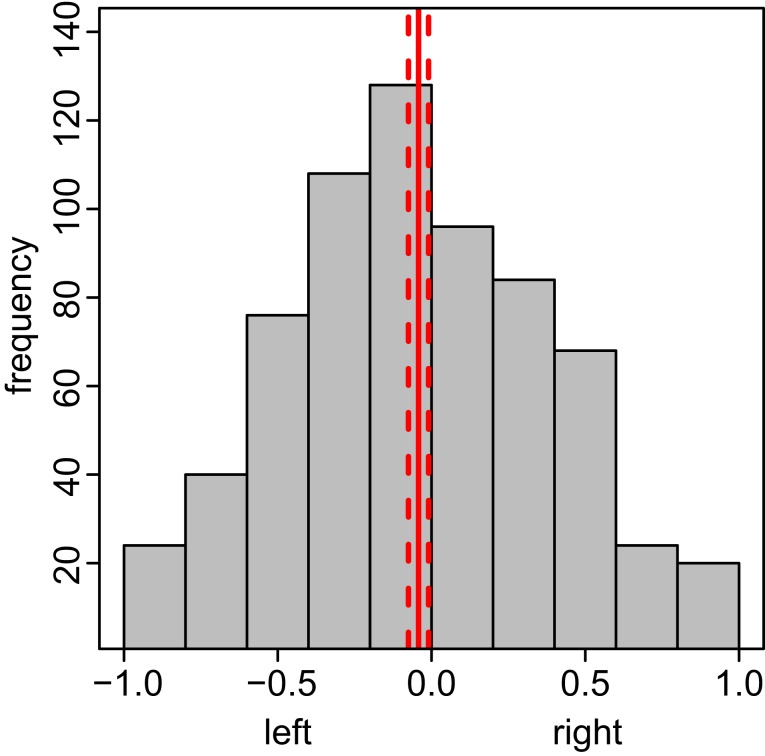
**Bees show a slight left preference in the conditioning chamber during test.** The x-axis depicts the side preference, ranging from −1 (complete left side preference) to 1 (complete right side preference), the y-axis the frequency with which the bees spent time at this side. Preference Indices calculated by taking the relative ratio between integrals of negative (left side movement) and positive (right side movement) positions over time for each bee (*N* = 174). The total test phase was used. Solid red line indicates the mean side preference; the dashed lines indicate 95% confidence interval.

**Figure A8:**
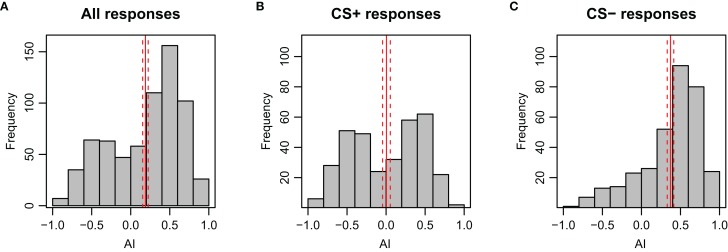
**Distribution of AI (Attractance Index) during stimulation period of the test phase for bees in APIS.** As expected, the AI is skewed to positive values during presentation of the CS−. The solid red lines indicate the mean side preference; the dashed lines indicate 95% confidence interval. **(A)** All responses included (*n* = 696, *N* = 174). **(B)** Only responses to the CS+ included (*n* = 348, *N* = 174). **(C)** Only responses to CS− included (*n* = 348, *N* = 174).

**Figure A9:**
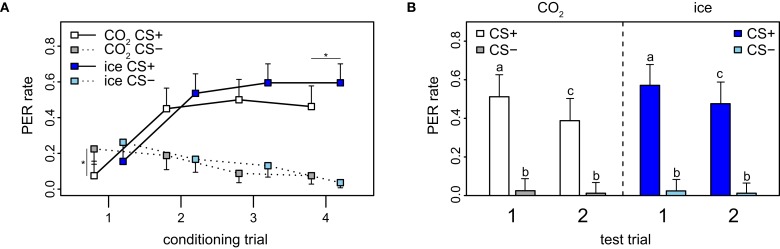
**Effect of anesthesia on conditioning and recall in PER. (A)** PER data stratified by anesthesia (CO_2_ and ice). The PER response was significantly lower for CO_2_-treated than for ice-treated bees for the last trial (*p* = 0.045, χ^2^ = 4.0, *df* = 1). Error bars indicate 95% confidence intervals and asterisks indicate significant differences (*p* < 0.05). **(B)** There was no significant difference between bees anesthetized with CO_2_ and bees anesthetized with ice for the test (*p* = 0.798, χ^2^ = 4.6, *df* = 8). Error bars show 95% confidence intervals, different letters indicate significant differences.

Bees showed a slight tendency for the left side in the conditioning chamber (Figure A7), the reason for this remains unclear, and we assume that slight differences in the light regime above the conditioning chamber due to asymmetric room illumination might have caused this bias. However, escapes as well as velocity and AI remain unaffected by this bias during conditioning and testing.

## Discussion

The investigation of learning and memory in invertebrates allows deeper insights into functions and mechanisms of smaller and less complex brains and thus paves the path for a better understanding of similar mechanisms in higher, more complexly organized organisms (Sattelle and Buckingham, 2006; Clarac and Pearlstein, 2007). Among invertebrates, the honey bee is one of the most prominent model organisms (Menzel, 1983), and honey bee conditioning is an important method to investigate learning and memory (Giurfa and Sandoz, 2012). We developed a novel approach to automatize aversive honey bee conditioning and compared it with classical appetitive PER conditioning. We chose to compare the results from APIS with PER instead of with SER because the former is the more widespread and better characterized conditioning paradigm for honey bees (Matsumoto et al., 2012). We were able to show that this new conditioning paradigm provides, for aversive learning, performance that is comparable with that from PER-conditioning for appetitive learning, offering a new tool for honey bee learning and memory research.

APIS combines the advantages of a controlled environment as used in PER/SER-studies with the opportunities of behavioral analysis of movements of an unrestrained animal, leading to response rates comparable with PER: both systems resulted in response rates of approximately 54%. Regardless of this conspicuous similarity it is also noticeable that our PER-response rates were relatively low compared to other studies (for an example, see Matsumoto et al., 2012). This might be explained by the low number of massed training trials and the very low odor concentration of only 10^−3^. Both have been previously shown to influence learning in honey bees (Menzel et al., 2001). Additionally, all the comparisons that were done here related to short-term memory, while mid-term memory and long-term memory remain to be investigated in APIS. Nevertheless, APIS is superior to PER-conditioning with respect to the number of bees which could be analyzed: over 90% of bees caught and put into the box could be used, whereas only 78% of the bees caught for the PER-conditioning went into the analysis. This difference can be explained by the more natural context: bees were caught at a feeder, transferred to the conditioning chamber and immediately trained and tested, whereas for PER, they had to be anesthetized, put into their harnesses and left for 2–3 h prior to the experiment.

### Bees modulate their escape behavior distinctly

The escape rate of the bees provided a binary (response/no-response) behavioral measurement similar to the extension of the proboscis in PER-conditioning. For PER-conditioning, a quasi-continuous physiological readout can be obtained with electromyograms of the muscle M-17, which is involved in proboscis extension (Smith and Menzel, 1989). APIS, allows the analysis of velocity and response-magnitude (AI) as behavioral continuous variables, giving access to more powerful analyses. For example, we found that the bees discriminated between CS+ and CS−, even when they appeared to escape from both (Figures 3B,C): velocity and AI were less negative for the CS− than CS+. These results imply that even if bees decide to escape from the safe odor (generalization), they respond less to the CS− than to the CS+. In APIS, the behavioral modifications we observed could be due to an operant learning component. Even though an escaping bee could not alter the shock stimuli, the protocol was designed so that most bees would experience that the shock ended promptly after it escaped from the odor injected side. In a free walking arena, it is impossible to eradicate all elements of operant learning, and this must be taken into consideration when interpreting the comparative results of harnessed and free walking paradigms. Additional experiments employing purely operant conditioning (e.g., where escape behavior induces shock stimulus termination) will have to be carried out in order to investigate the impact of any operant element.

### Better safe than sorry—generalization in APIS

We found a high response toward the CS− during the test (Figure 2A): 17.8% of the bees responded to the first CS− in APIS, whereas only 3.0% responded to the first CS− in the PER-conditioned bees. Responses to the CS− reflect generalization. Whereas most investigations of generalization focus on the nature of the CS (Ghirlanda and Enquist, 2003), our results suggest that generalization also depends on the nature of the US: aversive stimuli lead to a considerably higher degree of generalization than appetitive stimuli. Such an alteration of behavior might serve as a protective adaptation to noxious stimuli.

Generalization was asymmetric for PER (as observed in other studies, Bhagavan and Smith, 1997; Guerrieri et al., 2005) and APIS. In PER-conditioning, more bees generalized from nonanol to linalool than the other way around, while in APIS the situation was that more bees generalized from linalool to nonanol (Figure A1B). Specifically, AI and velocity were lower for the first linalool test trial in bees that received nonanol as CS+ compared to reversed odor configuration (Figures 3A,B). Both PER and APIS results would propose that the bees have a higher innate preference for linalool than for nonanol, i.e., linalool has a more positive hedonic value than nonanol. Indeed, linalool is a common floral odor that bees are likely to experience together with nectar rewards in nature, whereas 1-nonanol is found only in a few plants rarely visited by bees (Knudsen et al., 1993), and the structurally isomeric 2-nonanol is known to be one of several components of the bees' alarm pheromone (Collins and Blum, 1982).

### Extinction in APIS

When bees are exposed to repeated presentations of the CS without US, the conditioned response to the CS decreases steadily, a process called extinction (Sandoz and Pham-Delegue, 2004; Stollhoff et al., 2005; Eisenhardt and Menzel, 2007). Additionally, fatigue occurs, where also a decrease of conditioned responses is noticeable, but this process—unlike extinction—is not related to memory dynamics.

Unlike for PER, in APIS the escape responses did not drop significantly from the first to the second non-reinforced test trial of the CS+ (Figure 2A). However, extinction was visible for velocity and AI (Figures 3E,F). This reduction cannot be explained by exhaustion, because there would have been also a reduction in the speed with which the bees respond to the CS−, which does not occur. We propose that extinction for aversive stimuli occurs at a much slower rate than for appetitive stimuli in order to effectively avoid noxious stimuli (see above). Because they seem to act in a “better safe than sorry”-manner with respect to the generalization of odors, it would make sense to also follow the rule “once bitten, twice shy” when odors are repetitively given. This could be vital for honey bee biology and ecology: it is more advantageous to adapt more or less quickly to a decrease in volume and/or sucrose content as compared to adapting to a dangerous and possibly life-threatening situation such as an attack by a spider. Such an attack does not need a quick change in response but rather a long-lasting behavioral adaptation. Bees that experience crab spider attacks then avoid flowers containing these spiders (Dukas and Morse, 2003; Abbott, 2006; Jones and Dornhaus, 2011).

### Automatic conditioning in honey bees

Several attempts have been made to automatize honey bee PER-conditioning, the most important one by Vareschi (1971), who developed an apparatus for an automated PER conditioning. Though his attempt was successful and he was able to condition bees with his “Testautomat,” it was not reproduced until much later by Abramson and Boyd (2001). Presumably, an automated PER conditioning device has to face some restraints by honey bee morphology (e.g., proboscis length and antennal movement) which overstrains most automated systems. Also free-flying bees have been subjects of automation attempts (Núñez, 1970; Pessotti, 1972; Grossmann, 1973; Sigurdson, 1981; Abramson, 1986), and only recently, Abramson and co-workers developed a computer-controlled Skinner box for honey bees (Sokolowski and Abramson, 2010). Nevertheless, none of those different attempts was really coopted by the honey bee community, which is especially surprising since automated conditioning is a standard procedure in *Drosophila* research (for example the devices shown by Tully and Quinn, 1985; Putz and Heisenberg, 2002; Brembs, 2008; Claridge-Chang et al., 2009). Agarwal and co-workers recently adopted the idea of bees moving in a box on an electric grid as used to condition *Drosophila*, but their system still depends on an experimenter observing the bees' behavior or analysing the video recorded during the course of the experiment (Agarwal et al., 2011).

APIS therefore is the first system in honey bee research using freely moving animals in a controlled environment (the conditioning chamber) which combines fully automated conditioning as well as data acquisition and analysis. The response of bees to the odors was recorded automatically and analysed by a computer program, leading to a high degree of standardization, thus allowing a better comparison of honey bee performance between experiments within and across laboratories studying honey bee behavior. In future versions of APIS additional parameters might be quantified, such as reaction time and turning speed. As described above, we noticed a buzzing response during shock and also during the test when the CS+ was presented: a small microphone could be added to quantify this response. These parameters would quantify and describe the behavioral responses in detail.

The main findings of this study were that honey bees which were aversively conditioned in APIS escaped from the side where the shock-paired odor was given with an increased velocity and magnitude compared to an odor not paired with shock. In a short-term memory test bees conditioned in APIS or PER responded with comparable rates with respect to the conditioned odor. Secondly, bees modulated their behavior to repeated stimuli of aversively learned odors by reducing rate, speed and magnitude of escapes. Finally, our results suggest that generalization of two odors with different hedonic values might be affected differently by appetitive and aversive learning. Further investigations are needed to confirm the latter, because alternative explanations such as differences in anesthesia might also affect the bees' responses to odors during a recall test.

Additional experiments are also needed to assess possible interactions between classical conditioning and operant conditioning and to develop APIS into a setup that allows pure operant conditioning in a controlled environment, thus closing a gap currently existing in the investigation of honey bee learning and memory (Brembs, 2003). Furthermore, presently we cannot be certain what bees actually learn in APIS: our results suggest that they learn to associate the CS+ with the electric shock, but they might also learn that the CS− denotes the absence of electric shock. Similarly, how they react to a completely novel odor during test remains to be evaluated.

In conclusion, by providing a flexible yet standardized method APIS can hopefully supplement the current methods, and provide novel insight into learning mechanisms of honey bees.

### Conflict of interest statement

The authors declare that the research was conducted in the absence of any commercial or financial relationships that could be construed as a potential conflict of interest.
